# Residual TRM-to-Concrete Bond after Freeze–Thaw Cycles

**DOI:** 10.3390/ma14185438

**Published:** 2021-09-20

**Authors:** Paraskevi D. Askouni, Catherine (Corina) G. Papanicolaou

**Affiliations:** Department of Civil Engineering, University of Patras, 26504 Rio Patras, Greece; kpapanic@upatras.gr

**Keywords:** textile reinforced mortar (TRM), concrete, bond, normal-weight/lightweight matrices, freeze–thaw cycles

## Abstract

In the present work, the effect of various freeze–thaw cycles (namely, 0, 10, 30, 50, 60, and 70) on the residual bond characteristics of textile reinforced mortar (TRM)-to-concrete was experimentally examined. The TRM consisted of a carbon dry fiber textile embedded in a cement-based matrix. Two mortar types were used as the matrix: a normal-weight and a lightweight one sharing the same hydraulic powders but different aggregates (limestone and pumice sand, respectively). The single-lap/single-prism set up was applied after the specimens underwent hygro-thermal treatment (according to ASTM C 666-Procedure B). Failure was due to the sleeve fibers rupturing the load aligned yarns or textile slippage from the mortar for an exposure period ranging between 0 and 60 cycles and to TRM debonding from the substrate for 70 cycles. Increasing cycles resulted in the intensification of partial interlaminar debonding phenomena and the weakening of the textile-to-matrix bond, with lightweight mortar being more prone to these effects. In the absence of a commonly accepted standardized method for the assessment of the freeze–thaw resistance of cement-based composites, the criterion for the termination of the freeze–thaw sequence was the number of cycles inferring a shift in failure mode (from fiber rupture/fiber slippage to TRM debonding from the substrate).

## 1. Introduction

Textile reinforced mortar (TRM) is an innovative composite material consisting of inorganic matrices reinforced with fibrous technical grids (textiles). The textiles are made of carbon, glass, basalt, aramide, or PBO fibers that are usually arranged in two orthogonal directions; however, there are also hybrid grids with different types of fibers in each direction. Regarding the matrix, this can be a cement-, lime-, pozzolana- or geopolymer-based mortar (or any blend thereof), which can also be modified with polymeric additives. The field of TRM applications encompasses both new structures (e.g., prefabricated structural elements, stay-in-place formworks) and existing ones (strengthening of structurally deficient members). For the case of strengthening in particular, TRM-based solutions have proven their efficiency through numerous experimental studies and have already found their way onto the market. Depending on the specific set of parameters in effect (type of member to be strengthened, type of substrate, mortar, textile, number of textile layers, textile configuration and others), different degrees of strengthening efficiency may be achieved. Nevertheless, for all cases, the most decisive parameter governing the effective use of this family of composite materials in strengthening applications is the bond capacity of both the TRM-to-substrate and the textile-to-matrix interfaces ([1,2]). This bond capacity is also a function of the long-term performance characteristics of the composite, the TRM-protected substrate, and the interaction between them. The durability aspects of TRM composite materials externally bonded on existing structural members have not been extensively studied as of yet. Exposure cases of concern include (but are not limited to) freeze–thaw or wet–dry cycles, fire/high temperatures, and chemical attacks [3]. Therefore, an investigation of the effects of various aggressive environments on the bond performance of TRM strengthening systems is of high priority so as to ensure their prolonged and useful life.

The current paper focuses on the effect of freeze–thaw cycles on the bond response of TRM systems applied to concrete substrates. The existing studies on the topic are scarce and are briefly reviewed below. The authors of [4] studied the effect of chloride freezing-and-thawing cycles on the TRM-to-concrete bond through double-sided shear tests. They used a hybrid textile made of carbon and glass yarns in the warp and weft direction, respectively, which was impregnated with epoxy resin and was sprinkled with sand grains. The matrix consisted of a self-consolidating cementitious mortar. The examined parameters were the number of freezing-and-thawing cycles (0, 40, and 60 cycles), the pre-cracking degree of the TRM overlays, the addition of short-cut fibers in the matrix as well as the compression strength of the concrete substrate and its surface treatment. Each cycle had a circulating temperature range from +6 °C to −18 °C and a duration between 2 and 4 h while the freezing–thawing process was conducted after the specimens had been immersed in a NaCl solution with a 5% concentration for 24 h. The main conclusion was that the chloride freezing-and-thawing cycles did not seriously deteriorate the integrity of the TRM overlay but that they negatively affected the bond of the TRM-to-concrete interface. It was also concluded that the shear capacity of that interface could be improved by increasing the concrete strength, roughening the substrate’s surface, and adding short-cut fibers in the matrix, while the pre-cracking of the TRM overlays was also found to be beneficial to a certain extent. Ref. [5] studied the effect of heating and freezing cycles on the bond response of two TRM systems when applied on concrete masonry units using the single-lap/single-prism set-up. TRM systems consisted of dry PBO or carbon fiber textiles embedded in cementitious mortars. First, specimens were subjected to 50 freeze–thaw cycles; second, they were subjected to 150 high temperature cycles (from 27 °C to 50 °C); and third, they were re-subjected to 50 freeze–thaw cycles. Each freeze–thaw cycle consisted of freezing at −17.8 °C for 50 min and thawing at +4.4 °C for 50 min, with a transition period of 30 min. According to the results, the effectiveness of both textiles to resist the applied pull-out load was not significantly affected by either heating or freezing. However, even if it was not highlighted by the researchers, the textile slip at failure was significantly increased for treated specimens in comparison to the reference (non-treated) ones.

To the best of the authors’ knowledge, there are no other studies that examine the effect of the freeze–thaw cycling on the TRM-to-substrate bond; however, there are some studies investigating the tensile response of TRM coupons after their exposure to this type of environment. Ref. [6] investigated a TRM system made of cement-based mortar and a coated AR glass fiber textile. Both un-cracked and pre-cracked specimens were treated under various numbers of freeze–thaw cycles according to Procedure A of the ASTM C 666 standard [7] (temperature range: from 4 °C to −18 °C, cycle duration: 5 h). The tensile response of the specimens was characterized by: (i) damage due to the freeze–thaw cycles on the surfaces of the specimens that were free during casting and (ii) matrix self-healing and late hydration due to the permanence of the specimens in water on the surface that was in contact with the formwork during casting. The latter phenomenon was enhanced in the case of the pre-cracked specimens due to the presence of cracks, which facilitated the water penetration. Additionally, it was concluded that the examined TRM presented low sensitivity to freezing–thawing cycles. Ref. [8] studied the effect of various aggressive environments on the tensile response of a TRM system made of a cementitious matrix and one or two layers of coated glass yarn textiles with three different square weights. Among the examined environments, namely the alkaline environment, the acidic environment, and the freeze–thaw cycling environment (frosting at −18 °C for 4 h and defrosting at 20 °C for 2 h), it was found that the last one had the most negative effect on the tensile properties of the specimens since it caused the formation of micro-cracks. The self-healing of the matrix due to the freeze–thaw process was observed in that study as well and was attributed to the surplus of the active mineral additive that was added to the mortar. Finally, it was concluded that the resistance of the studied TRM system to the aggressive environment increased with the increase of the applied reinforcement ratio. Ref. [9] studied a TRM system comprising one carbon fiber textile strip impregnated with organic adhesion promoter and a semi-hydraulic lime mortar as the matrix. Among various aggressive environments, i.e., alkaline, saline, distilled water, hydrochloric acid environments, freeze–thaw cycling (freezing at −18 °C for 4 h followed by exposure to 37.7 °C and 100% relative humidity for 12 h) was also examined, and it was found that it offered a small enhancement to the tensile performance of the composite. Ref. [10] prepared a TRM system that consisted of two AR glass polymer coated textile strips sandwiched between three layers of cementitious based mortar. They also studied various environmental loading types including freeze–thaw cycling, which was conducted based on the NBN EN 12467 Standard [11] (lowering the temperature from +20 °C to −20 °C repetitively). The major conclusion of the study was that the initial stiffness of the specimens was reduced by a minimum 60% due to microcrack formation, while their post-cracking stiffness was not decreased since the textile fibers were unaffected. Based on the aforementioned studies—although they are limited—it can be noted that due to different boundary conditions, the TRM coupons constructed for tension testing were found to be prone to cracking after their thermal treatment while composite integrity was not seriously affected in cases where it was applied as an external overlay. Nevertheless, the self-healing phenomenon—which is related to the structure of a microscopic composite and not to specimen from—was mentioned or even implied irrespectively of the test type.

With the exception of residual bond and tension capacities, some other mechanical characteristics of the TRM were also examined after or while the composite was being exposed to freeze–thaw treatment. Ref. [12] investigated the effect of chloride freeze–thaw cycles on the yarn-to-matrix bond capacity and on the flexural performance of thin plates concerning the TRM system described in [4]. The parameters under consideration were the number of freeze–thaw cycles (temperature range: from 5 °C to −18 °C, duration: 3 h) and the addition of different types of short-cut fibers in the matrix as well as the effect of various sustained bending loads during cycling. According to the experimental findings the increase of freeze–thaw cycles did not significantly affect the interfacial yarn-to-matrix bond capacity in contrast to the TRM plates’ bending capacity, which was noticeably degraded. Furthermore, the presence of fibers was either not important or negative, depending on their type. Finally, it was concluded that the combined effect of chloride freeze–thaw cycles and sustained bending load accelerated the deterioration of the TRM flexural performance with respect to the effect of freeze–thaw cycling only, while the deterioration degree of the flexural performance increased as the sustained load increased.

Finally, the study of [13] is mentioned, as it may be the only one that concerns the effect of chloride freeze–thaw cycles on a structural member strengthened with TRM. The control specimen—a RC beam strengthened with a double layer of the TRM system used in [4]—was subjected to 40 freeze–thaw cycles (temperature range from 5 °C to −18 °C at the center of the specimen and a duration 4 h), and its bending capacity and mid-span deflection were compared to those of respective specimens subjected to the same number of cycles and the simultaneous effect of various sustained bending loads. All of the specimens underwent shear failure due to concrete strength loss caused by the freeze–thaw cycling, while the TRM overlay retained its integrity and its bond with the concrete substrate protecting the bending section of the beams. According to the results, as the sustained load increased, the bearing capacity decreased, and the maximum crack width and deflection increased since the damage rate of the concrete was accelerated during the freeze–thaw cycles.

This brief literature review cannot lead to generalized conclusions neither in the case where TRM is examined as external overlay of an existent substrate nor in the case that the tensile response of this composite is investigated. Due to the wide variety of the constitutive materials comprising TRMs, a larger database should be developed through future experimental work. Additionally, a commonly accepted standardized method has to be developed for the effect of freezing–thawing on the mechanical characteristics of this composite material. However, the aforementioned studies provide some positive indications in the direction of TRM application as a protective overlay for structural members that are subjected to freezing–thawing. In this context the present work aims to enrich the data base of the experimental results that concern the effect of this aggressive environment on the TRM-to-substrate bond.

## 2. Experimental Procedure

### 2.1. Materials

#### 2.1.1. Substrate

**Figure 1:**
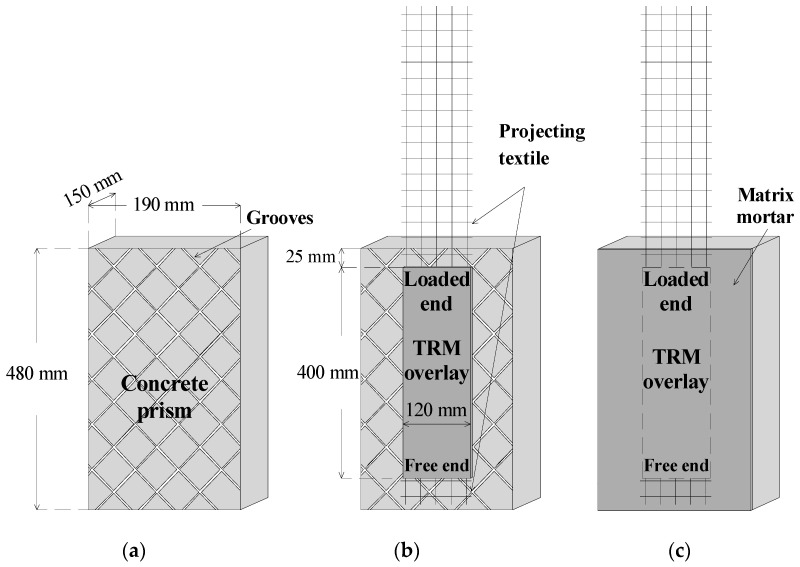
Different stages of specimen construction: (**a**) after surface treatment, (**b**) after application of the TRM strip, and (**c**) after complete coverage of its surface with mortar.

The concrete substrate was simulated by prisms with the dimensions of 480 mm × 190 mm × 150 mm, representing length × width × height. Prior to strengthening, the surfaces of all of the prisms were prepared by removing the laitance and by creating a grid of 3 mm-deep grooves with a handheld disc grinder (Figure 1a). The concrete mixture contained ordinary Portland cement (CEM II 32.5 N), limestone sand (*d_max_* = 4 mm), limestone gravel (*d_max_* = 15 mm), water, and an air entraining agent in the proportions (kg/m^3^) 340:900:850:175:2.04. The air entrainer was used in order to shield the substrate from the detrimental action of the freeze–thaw cycles and to render the TRM strip as the weak link of the assembly. The compressive strength of the concrete at 28 days was equal to 48 MPa.

#### 2.1.2. TRM

Two TRM systems were used and shared the same textile but different matrices. The reinforcement was a textile made of dry carbon fibers equally arranged in two orthogonal directions with a 20 mm mid-yarn spacing and an aerial weight of 170 g/m^2^. The textile’s tensile strength (*f_tex_*) and elastic modulus (*E_tex_*) were found to be equal to 839 MPa (CoV 6%) and 279 GPa (CoV 16%), respectively; these values were determined through the lab testing of six textile strips, each comprising five yarns and following most of the mandates of the EN ISO 13934-standard [14]. The same mechanical characteristics were determined after the insertion of six textile strips into the freeze–thaw chamber and after exposure to the maximum number of freeze–thaw cycles adopted in the current study (i.e., 70 cycles). All of the strips were tested in dry conditions and following the same experimental method and means as in the case of non-exposed strips. According to the visual inspection of the exposed strips, the latter seemed to remain unaffected by freeze–thaw action. The tensile strength of the freeze–thaw-stricken textiles was equal to 917 MPa (9% higher than the unexposed textile, which is well within the statistical variation of both samples). The respective modulus of elasticity was found to be equal to 246 GPa (12% lower than the unexposed textile, which is also well within the statistical variation of both samples).

Two cement-based mortars of equal compressive strength and different densities were used as matrices. The normal weight mortar (denoted as ‘N’, henceforth) contained Portland cement, fine limestone sand, silica fume, limestone filler, and water. The lightweight mortar (denoted as ‘L”, henceforth) comprised the same constituent materials, with the difference being in the sand, which came from pumice. The composition and the flow table test results of each matrix are provided in Table 1. The air-dry densities of the normal weight and lightweight mortars were equal to 2113 kg/m^3^ and 1760 kg/m^3^, respectively. The compressive and flexural strengths of the mortars were determined at 28 days, as per EN 1015-11 [15], and were found equal to 50 MPa (CoV 3%) and 5 MPa (CoV 15%) and 50 MPa (CoV 12%) and 3 MPa (CoV 17%) for mortar N and mortar L, respectively. It is noted that the mortar prisms used for the mechanical characterization of the matrices were cast using a part of the total mortar batches mixed for the cast of the TRM strips and that they were cured next to the reinforced concrete prisms.

**Figure 2:**
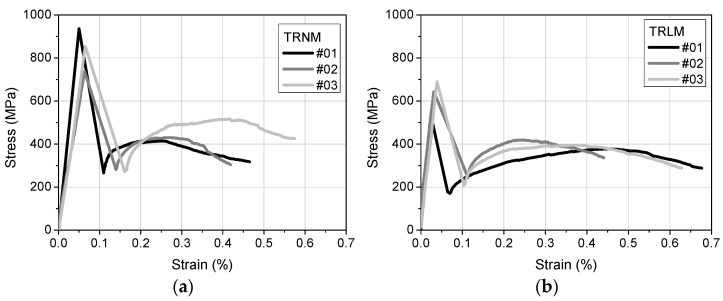
Axial tensile stress versus axial tensile strain curves of: (**a**) TRNM and (**b**) TRLM rectangular coupons.

It was decided to furnish the concrete prisms with single-layered TRM strips so as to minimize the number of components comprising the composites and, hence, to facilitate the interpretation of post-exposure phenomena. The tensile response of textile reinforced normal weight mortar (TRNM) and textile reinforced lightweight mortar (TRLM) rectangular coupons was experimentally assessed according to the procedure described in AC434 ICC-ES [17] by testing three identical specimens per TRM type. The derived axial tensile stress versus the axial tensile strain curves are depicted in Figure 2. As expected, the under-reinforced coupons responded in a manner that does not qualify as that of a strain-hardening material, exhibiting a substantial load drop after the first crack. This loss of load carrying capacity was irrecoverable and could be owed to the low volume fraction of the fibers and the inadequacy of the fibers to effectively bridge the crack. Failure occurred due to fiber slippage from the mortar combined with individual sleeve filament rupture, leading to the enlargement of the crack. The tensile strength (*f_TRM_*) and the corresponding axial strain (*ε_TRM_*) of TRNM were equal to 842 MPa (CoV 12%) and 0.06% (CoV14%), respectively, while the *f_TRM_* and *ε_TRM_* of TRLM were equal to 617 MPa (CoV14%) and 0.03% (CoV 20%), respectively.

### 2.2. Bond Specimens

Each bond specimen consisted of a concrete prism that was unilaterally furnished with a single TRM strip (Figure 1b), which was applied following a wet lay-up procedure. The TRM overlay comprised one textile layer sandwiched between two layers of mortar with a thickness of 4 mm each. The mortar layers were toweled flush to the prescribed thickness by means of a rubber cork frame. The TRM overlay (and hence the textile strip) of each specimen was centrally bonded on the prism width-wise, and its length and width were equal to 400 mm and 120 mm (5 yarns), respectively. The distance of the TRM overlay from the prism’s top edge was equal to 25 mm in order for stress concentration phenomena to be avoided. The textile projected from both sides of the TRM overlay, that is, from the top and bottom part of the overlay, named as “loaded end” and “free end”, respectively (Figure 1b).

Right after the TRM overlay was cast on the free part of the prism’s surface, it was covered with the same mortar used as the matrix in the composite (Figure 1c) in order to simulate a certain type of real-life conditions in which the full coverage of the structural member with the overlay is realized. In these cases (and assuming—ideally—a crack-free overlay), the TRM-to-substrate and the textile-to-matrix interfaces are not immediately exposed to freezing–thawing. Instead, a unidirectional (normal to the TRM-covered surface) attack front is established, which can be easily simulated by the specimens employed in this work. Had the shear bond specimens been constructed “as usual” (i.e., without full coverage by mortar) and subjected to freeze–thaw cycles, both the TRM-to-substrate and the textile-to-matrix interfaces could (and probably would) have been damaged along the perimeter of the TRM strip. This, in turn, could lead to premature strip debonding, thus masking the “net” contribution of the TRM overlay to the deterioration mechanisms.

The specimens were moist-cured for 7 days by being covered with wet burlaps and were then stored in lab conditions (20 °C ± 2 °C, 65% RH) for 21 days until the commencement of their hygro-thermal treatment. Mechanical (shear bond) testing took place 7 days after the end of the exposure period. In the meantime, the specimens were stored in the aforementioned lab conditions.

It should be noted that the choice of the TRM bond length was dictated by the requirement for the adequate anchoring of the textile within the mortar. The authors relied on the experimental results from previous studies concerning the in-plane shear response of single-layered TRM strips (bonded on concrete) comprising dry carbon fibers textiles embedded in cementitious matrices. In particular, Ref. [18] concluded that the effective bond length (i.e., the minimum length of the TRM overlay for which the bond load at the fibers-to-matrix interface maximizes) of a TRM system consisting of a textile sharing the same aerial weight and geometry as that used in the current study was less than 450 mm. According to the results of [1], the effective bond length of a TRM system comprising a textile with almost double the aerial weight and half the mid-yarn spacing from the one that was being currently used was found to be in the range of 200–300 mm. In the aforementioned studies, the determination of the effective bond length was based on the assumption that this was indicated by the onset of load stabilization on the curve of the maximum bond load versus the bond length [19]. In the study of [20], which involved a TRM system made of a textile identical to the current one, the effective bond length was determined based on the axial strain distribution along the textile, which was proposed to be between 200–330 mm. Given the above information, the authors made a conservative choice regarding the bond length so that the in-plane bond strength of the used composite material—in the absence of TRM/substrate debonding—could be fully exploited.

### 2.3. Hygro-Thermal Treatment

**Figure 3:**
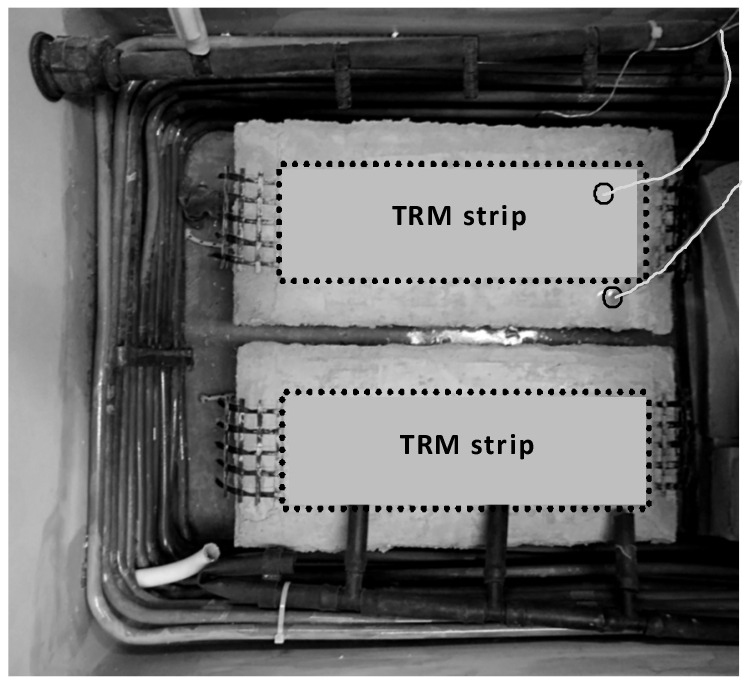
Two identical specimens inside the freeze–thaw chamber.

**Figure 4:**
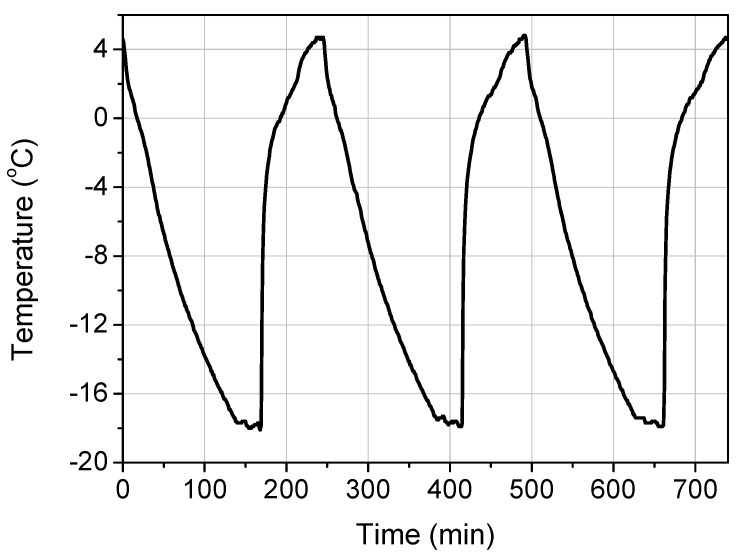
Temperature inside the TRM overlay versus time during freeze–thaw cycling.

After the end of a 28-day curing period (as previously described) and prior to mechanical testing, the shear bond specimens were subjected to varying freeze–thaw cycles in a chamber that was specifically designed to follow the thermal protocols described in Procedure B of the ASTM C 666 standard [7] (see Figure 3). Each cycle lasted 240 min (4 h) and consisted of a rapid freezing period (from +4 °C to −18 °C) in air with a duration of 165 min followed by a thawing heating period (from −18 °C to +4 °C) in water with a duration of 75 min. The temperature of the specimens was monitored through two K-type thermocouples. One was embedded in the concrete prism (at a depth of 60 mm from the TRM face) and served as the controlling sensor for chamber operation while the other was placed inside the TRM overlay (at a depth of 4 mm from its face). The maximum difference between these two temperature readings was found to be less than 1 °C during each freeze–thaw cycle. Figure 4 depicts the temperature recorded by the thermocouple that was inside the TRM versus time during three typical freeze–thaw cycles.

### 2.4. Shear Bond Test Set-Up

**Figure 5:**
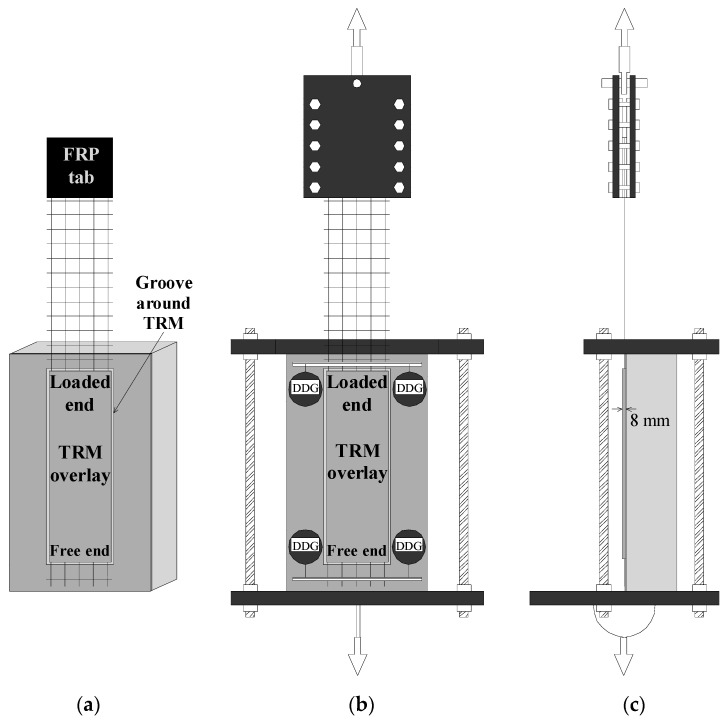
Specimen before testing (**a**), front (**b**) and side (**c**) view of the set-up.

Shear bond tests were conducted by partially following the single-lap/single-prism set-up as described in the recommendations of RILEM TC 250-CSM [21]. Prior to testing, the extremity of the projecting textile from the loaded end of the TRM overlay was sandwiched between two glued-on fiber-reinforced polymer (FRP) tabs. Additionally, a through-the-mortar groove was cut around the TRM overlay using a handheld diamond wheel in order to separate the overlay from the rest of the mortar covering the face of the concrete prism (Figure 5a). Each specimen was placed in a steel frame, which was adjusted in a servohydraulic testing machine with a load capacity equal to 250 kN. The projecting textile was connected to the fixed part of the machine through a joint that provided full in-plane and partial out-of-plane rotation capacity (Figure 5b,c) and was that pulled out from the matrix with a displacement rate equal to 0.005 mm/s. As also depicted in Figure 5b, the instrumentation of each specimen included (i) two digital dial gauges (DDG) attached to the wall close to the strip’s loaded end to act against an aluminum plate glued on the first transversal yarn of the pulled textile to record the textile’s relative displacement in respect to the concrete prism (under the assumption of zero TRM-to-substrate slip) and (ii) two DDG glued to the wall close to the overlay’s free end to act against an aluminum plate glued to the first transversal yarn of the projecting textile to record the textile’s slip (applied only on one specimen in a group of identical specimens).

### 2.5. Experimental Program

In total, 24 specimens were tested after being exposed to freezing–thawing. Half of the specimens (12) received TRNM overlays and were subjected to various numbers of freeze–thaw cycles, namely 0 (control specimens), 10, 30, 50, 60, and 70, using two identical specimens per number of cycles. The rest of the specimens (12) were reinforced with TRLM overlays and were exposed to the same program of freeze–thaw cycles. The notation of the specimens has the form of FTx_My_n, where x is the number of freeze–thaw cycles, y is the type of mortar used as matrix (N or L), and n is the specimen number in a group of identical specimens (see Table 2).

## 3. Results

### 3.1. Failure Modes

All of the TRNM specimens (except for the ones subjected to 70 freeze–thaw cycles), control TRLM specimens, and TRLM specimens subjected to 10 freeze–thaw cycles failed due fiber rupture. The failure of a critical amount of sleeve (well-bonded to mortar) fibers led to the loss of load-carrying capacity, as the inner (core/unbonded) fibers in the yarns that were left intact slipped through the mortar failing to result in strength recovery. The rest of the TRLM specimens (except for the ones subjected to 70 freeze–thaw cycles), failed due to the slippage of the textile from the mortar. This failure mode was also accompanied by the rupture of (a small number of) individual sleeve fibers. For all of the specimens, the TRM strips remained uncracked during testing. As explained below, the type of mortar and the number of cycles had an influence on the degree of damage evidenced after freeze–thaw exposure.

**Figure 6:**
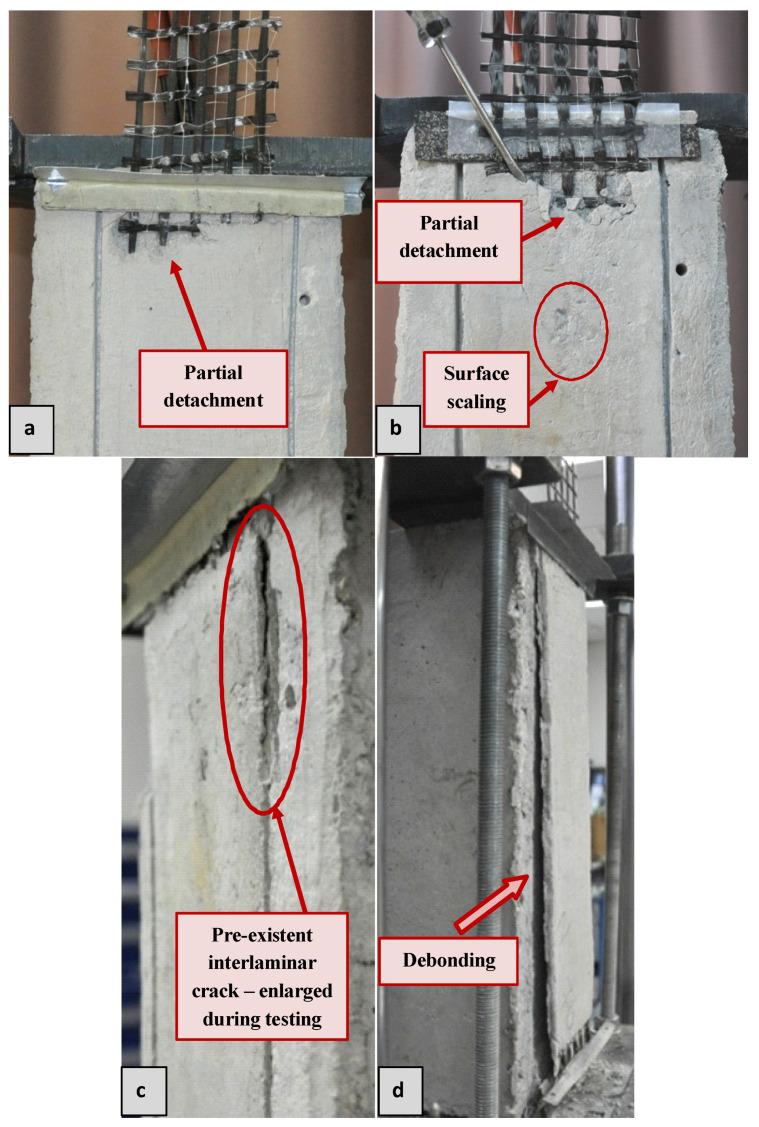
Post-failure photos of (**a**) FT10ML02, (**b**) FT30ML02, (**c**) FT50ML01, and (**d**) FT70MN01 specimens.

In the case of specimens subjected to 10 and 30 freeze–thaw cycles, post-exposure (and pre-test) integrity checks revealed partial detachment of the top mortar layer over a limited area close to the loaded end (Figure 6a,b). Integrity checks included the visual assessment of the strip’s surface and tapping it with the tip of a screwdriver. The production of a hollow sound signified the detachment of the top mortar layer from the rest of the composite. It should be noted that despite the partial detachment, the top mortar layer remained in place during the entire duration of shear bond testing. Photos in Figure 6a,b were taken after testing when the top mortar layer over the detached areas had intentionally (and effortlessly) been flaked-off using the tip of a screwdriver.

For the same number of freeze–thaw cycles, mortar detachment close to the loaded end (also present in specimens subjected to more than 30 cycles) was more extensive for the TRLM overlays than for the TRNM ones. In addition, surface scaling occurred over small areas sparsely distributed over the top mortar layer for all of the specimens that had been subjected to ≥ 30 cycles. Scaling was shallow (approx. 1 mm-deep); hence, the textile was not revealed in these areas (Figure 6b). The lightweight matrix was found to be more prone also to surface scaling than the normal-weight one.

Visual inspection of specimens that had been furnished with TRLM strips and that had been subjected to 50 freeze–thaw cycles revealed the formation of fine cracks along the top mortar layer-to-textile interface (interlaminar cracks) that were close to both ends of the TRM strip. The interlaminar cracks formed at the loaded end enlarged during shear loading and caused the increase of the detached area (see in Figure 6c photo taken post testing.

In the case of the specimens subjected to 60 freeze–thaw cycles, the aforementioned pre-existing interlaminar cracks were present for both types of TRM overlays and caused an increase of damage (top mortar layer detachment) in the vicinity of the load introduction. It is noted that the bottom mortar layer of all of the specimens subjected to ≤60 freeze–thaw cycles remained firmly bonded onto the substrate after testing.

All of the specimens subjected to 70 freeze–thaw cycles failed due to the TRM debonding from the substrate (Figure 6d). Failure was abrupt in the case of the TRNM strips causing the early termination of the test. On the contrary, the debonding of the TRLM strips occurred gradually during testing. It is noted that pre-test interlaminar cracks were also present after the specimens had been exposed to 70 cycles for both types of mortars. Post-test inspection of block surfaces revealed that all of the grooves were filled with mortar. The sheared-off TRNM strips were free of concrete remains, whereas small pieces of the latter were traced on the debonded TRLM strips. All of the observed failure modes are summarized in Table 2.

Finally, it is noted that visual inspection of the concrete prisms revealed that they did not suffer any damage, even after exposure at the maximum number of freeze–thaw cycles employed. The substrate remained intact (zero residues produced) due to the use of an air-entraining agent in the concrete mixture. The aim was to drive damage generation on or within the externally bonded composite strip, as it was its behavior under freeze–thaw cycling that was unknown.

### 3.2. Specimens’ Response

**Figure 7:**
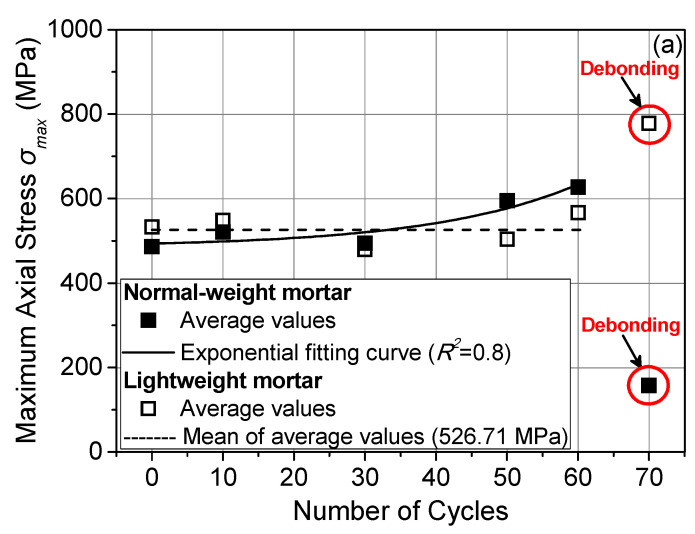

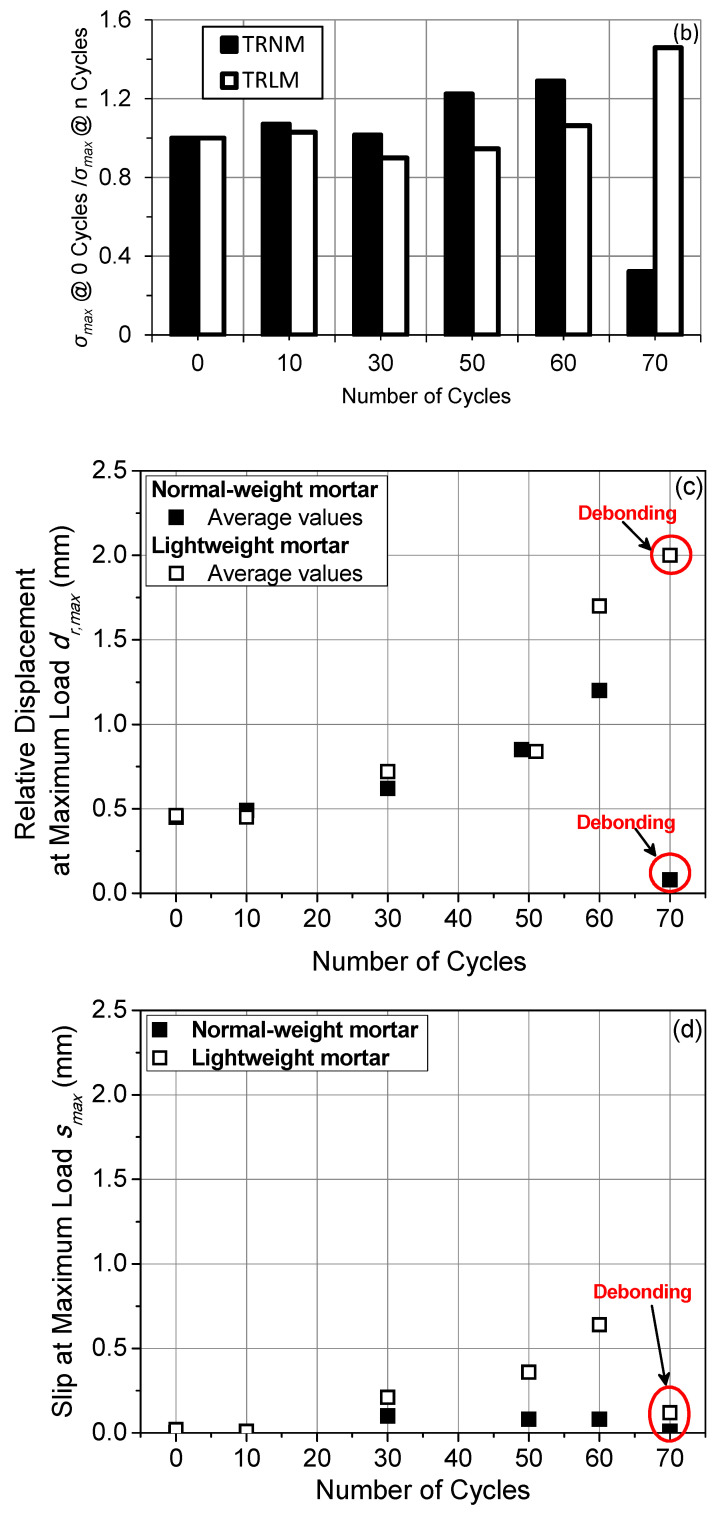
(**a**) Maximum textile axial stress (*σ_max_*), (**b**) ratio of *σ_max_* corresponding to the reference specimens to *σ_max_* of exposed specimens, (**c**) relative displacement (*d_r,max_*), and (**d**) slip (*s_max_*) corresponding to *σ_max_* versus number of freeze–thaw cycles.

The test results are presented in Table 2 are based on the following parameters: (i) maximum textile axial stress (*σ_max_*) computed by dividing the maximum load carried by the TRM overlay with the cross sectional area of the longitudinal (load-aligned) fibers, which is equal to 4.80 mm^2^, (ii) the relative displacement of the textile with respect to the wall (*d_r,max_*—corresponding to maximum load) being equal to the average of the readings from the DDG at the loaded end, and (iii) the textile slip from within the mortar (*s_max_*—corresponding to maximum load) being equal to the average of the readings from the DDG at the free end. The average values of *σ_max_* and *d_r,max_* and the value of *s_max_* versus freeze–thaw cycles are depicted in Figure 7a,c,d, for both types of TRM. In addition, the *σ_max_* values shown in Figure 7a are presented in Figure 7b and are normalized over the average *σ_max_* value corresponding to the reference (control) specimens.

The average values of *σ_max_* corresponding to the TRNM specimens display a slight exponential increasing trend as the number of freeze–thaw cycles increased from 0 to 60 cycles (Figure 7a). Nevertheless, the difference between *σ_max_* after 0 freeze–thaw cycles (control specimens) and that after 10 and 30 cycles is <10% (Figure 7b), which is comparable to the CoV values of *σ_max_* (≤10%) for each TRNM specimen group failing due to textile slippage (excluding group FT10MNi). In the case of TRLM specimens, the average values of *σ_max_* do not follow the aforementioned exponential trend with increasing freeze–thaw cycles up to 60, but they fluctuate around a mean (maximum difference between average *σ_max_* values ≤ 10%, Figure 7a). The CoV values of *σ_max_* for each TRNM specimen group—without exceptions—are less than 10%. It is also noted that the *σ_max_* values for the specimens subjected to the same hygro-thermal regime (and up to 60 freeze–thaw cycles) are comparable for specimens with different types of TRM strips (different mortars); the maximum difference is, in general, to the order of 10% (18% for specimens subjected to 50 cycles—Figure 7b).

The average values of *d_r,max_* for both types of specimens follow an exponential increasing trend with increasing freeze–thaw cycles up to 60 (Figure 7c). As discussed in a previous work by the authors [18], if each longitudinal yarn is considered as a solid assemblage of fibers, then it can be assumed that the recorded *d_r_* values are the combined result of yarn elongation and of their slippage(s) from within the matrix under the condition that the relative displacement of the TRM overlay in respect to the substrate is zero during shear bond tests. The *s_max_* values for the reference specimens and specimens subjected to 10 freeze–thaw cycles (regardless of mortar used) are close to null. For the TRNM specimens, the *s_max_* values are very low and do not vary significantly within the range of 30 to 60 cycles (Figure 7d). The respective values for the TRLM specimens are higher than those for the TRNM specimens and increase exponentially with an increasing number of cycles (Figure 7d). In light of the above observations, it can be concluded that for all of the specimens excluding the TRLM ones subjected to 30, 50, and 60 freeze–thaw cycles (i.e., the ones suffering heavier damage), the dominant component of *d_r,max_* is the elongation of the textile (i.e., the fiber strain at maximum load multiplied by the textile unbonded length). The latter implies good matrix-to-textile bond conditions over a large portion of the bonded area despite the fact that this portion decreases (slightly) with increasing cycles of exposure. Larger slip values are associated with heavier (more extensive) damage in the composite, which are manifested in this work as the partial debonding of the top mortar layer and interlaminar cracking. The increase of the *d_r,max_* values in this case is owed to the increase of both components (textile elongation and slip).

The abrupt debonding of the TRNM strips from prisms subjected to 70 cycles occurred at a slip close to zero. In the case of the TRLM strips, the debonding from the substrate was gradual, as previously described (see Section 3.1). Consequently, in this case, *d_r,max_* comprises yet another component of the slip of the entire composite relative to the substrate. However, the latter was not monitored during testing. It is highlighted that all comments related to the *d_r,max_* and *s_max_* values are of a more qualitative value due to the inherent variability associated with the respective measurements.

**Figure 8:**
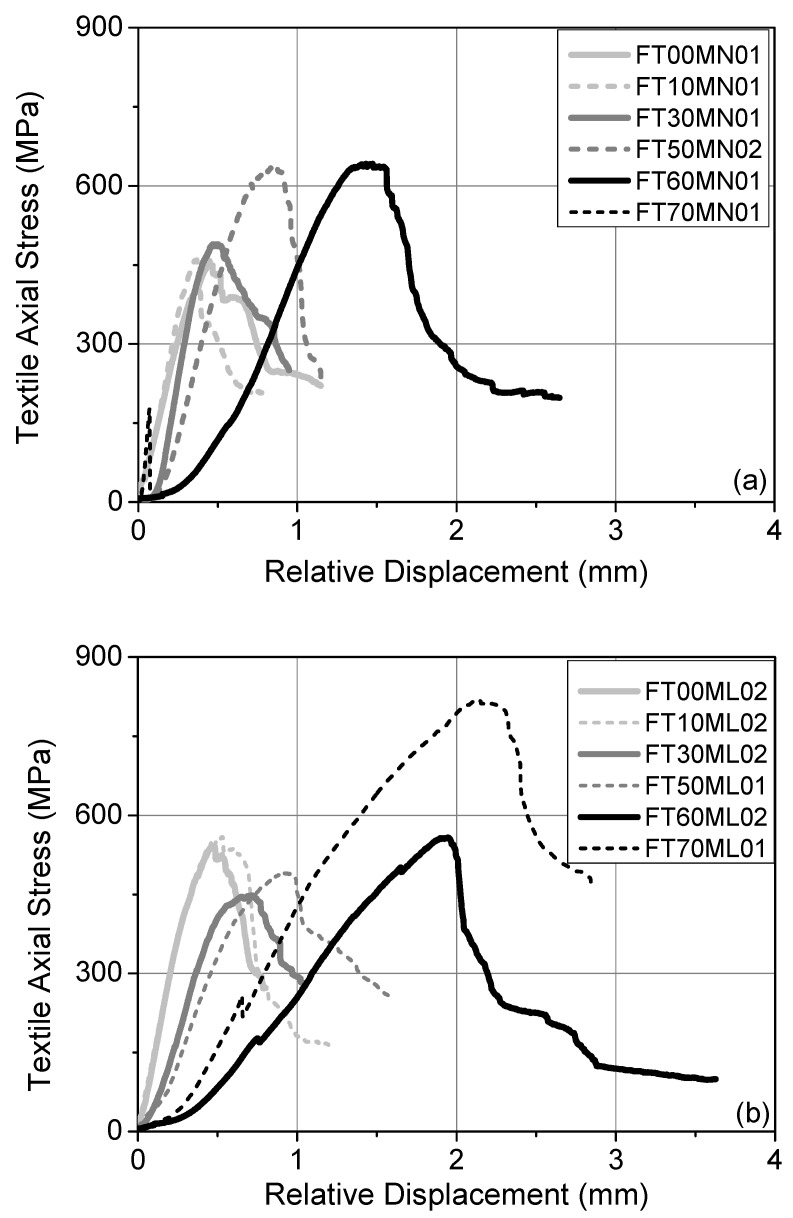
Response curves (*σ* vs. *d_r_*) of representative specimens furnished with (**a**) TRNM and (**b**) TRLM overlays.

In Figure 8, the response curves of the representative specimens furnished with TRNM (a) and TRLM (b) overlays are depicted in terms of textile axial stress (*σ*) versus the relative displacement of the textile with respect to the wall (*d_r_*). It is noticed that the type of mortar does not affect the shape of the curves in cases where they share the same exposure protocol and the same failure mode. Depending on the failure mode (i.e., whether fiber rupture preceded textile slip or not), the ascending branch of the response curves is either quasi-linear or deviates from linearity and is close to maximum. A quasi-linear ascending branch corresponds to almost full composite action, whereas a non-linear one denotes that the yarns-to-matrix bond is gradually deteriorated along a part of the bond length (due to slipping). As the freeze–thaw-induced damage increases, the following observations apply:

(i) The “stiffness” of the TRM (i.e., the inclination of the ascending branch) reduces the signification of the compromised textile-to-matrix bond conditions, which is mainly due to the partial debonding of the top mortar layer in the vicinity of the loaded end. A “stiffness” decrease with increasing freeze–thaw cycles is more pronounced in TRLM (damage-prone) specimens.

(ii) There is a certain “drag” in the curves, that is, a delay in the *σ* increase with when the relative displacement increases at the onset of the test; this delay is due to the secondary freeze–thaw-induced effect, that of the interlaminar cracks (namely, those close to the loaded end). In its post-exposure condition, the textile had a larger free length than the one that it was initially rendered with and remained non-activated until the relative displacement reached a value that was adequate to stretch it.

After the attainment of maximum load, the load distribution among the longitudinal yarns does not remain uniform. Therefore, the level of the residual load plateau varies depending on the number of load-aligned fibers remaining intact. The nature of this phenomenon is highly stochastic (especially for single-lap/single-prism shear bond test setups and for textiles with dry fiber yarns), resulting in large coefficients of variation within a test group of identical specimens.

The response curve representative of the specimens furnished with TRNM overlays and that were subjected to 70 freeze–thaw cycles consists of a short linear ascending branch followed by a sharp load drop, depicting the abrupt debonding of the TRM strip from the concrete face. Despite the fact that the specimens with TRLM overlays shared the same failure mode, their representative response curve resembles those of specimens exposed to less than 70 cycles. It is also worth noting that the efficiency factor for this specimen group (*σ_max_*/*f_tex_*) is equal to 0.93. Additionally, the curve exhibits larger “stiffness” and shorter drag in respect to the FT60ML02 curve. Note that in this case, the relative displacement also contains the TRM slip component relative to the prism. An attempt to provide reasoning for this behavior is provided in Section 4.

## 4. Discussion

Observations based on post-exposure (and pre-test) integrity checks led to the identification of two damage paths: one through the textile protruding from the mortar both at the loaded and the free end and another through the top mortar layer. The role that these paths play in the freeze–thaw deterioration process is analyzed further on.

According to [6], the projecting textile at both ends of the composite and especially its longitudinal yarns can act as water transfer channels that facilitate water penetration in the matrix. It is logical to assume that this water transfer capacity increases with a decreasing degree of filament impregnation with a binding means during the construction of the textile; hence, this this is the maximum capacity for dry fiber bundles. This water transfer channeling seems to be the main cause for the partial debonding of the top mortar layer close to the edges of the strip (see also Section 3.1). Critical for the composite’s behaviour is the top mortar layer debonding in the vicinity of the load introduction line. It should be noted that the protrusion of the fiber yarns was not only dictated by the mandate of the specific shear bond test recommendation followed in this work (RILEM TC 250-CSM recommendation [21]) but also by the necessity for easy specimen handling and the space limitations of the freeze–thaw chamber employed.

In real-life applications, exposed fiber yarns—similar to those on either side of a crack—can act as water transfer channels and can result in the same type of top mortar layer debonding. Crack widening due to repetitive freeze–thaw action (under the assumption that the crack remains in a water-filled state during thawing) can even cause damage to the fibers.

Focusing on the experimental results produced in the framework of the present work, the extension of the debonded zone in the top mortar layer was not large enough to reduce the available bond length to a size lower than the effective one. The maximum length of this zone was recorded for the case of FT60MLi specimens and was approximately equal to 25% of the bond length. The remaining sound part of the bond length appears to be within the proposed range of the effective bond length, as presented in Section 2.2; hence, at the start of each shear bond test, the full bond capacity of the TRM overlays was available.

Water infiltration through the longitudinal fiber yarns can also be considered as the main mechanism behind the formation of interlaminar cracks along the top mortar layer/textile interface. Water gradually propagates from the longitudinal yarns to the transversal ones, reaching the side faces of the TRM strip.

The second damage path is through the top mortar layer. Freeze–thaw cycling causes both superficial damage (surface scaling) and in-depth deterioration (degradation of the TRM-to-substrate bond, which results in specimen failure after being subjected to 70 freeze–thaw cycles). The aforementioned damage processes did not have an adverse effect on the *σ_max_* values for all of the specimens subjected to up to 60 freeze–thaw cycles. On the contrary, for increasing freeze–thaw cycles, the *σ_max_* either increased or remained constant in the TRNM and TRLM series, respectively. A possible explanation for this phenomenon can be found in the late hydration of the mortars, as further discussed below.

Late hydration during the repetitive freeze–thaw exposure of TRM composites has also been reported from previous studies, such as those of [4,6,8]. The presence of micro cracks before the exposure of a TRM system to freeze–thaw cycling and/or their formation during this thermal treatment facilitates water penetration in the matrix, promoting self-healing and fiber-to-matrix bond improvement. Hydration products can also form within the fiber yarns, increasing the ring area of well-bonded filaments.

During freeze–thaw exposure, the effects of late hydration are in a dynamic antagonism with those of frost heaving. Different types of cement-based mortars respond in a distinctively different manner to this condition. The complex phenomena in action can only be investigated using microstructural damage detection techniques, such as optical microscopy, scanning electron microscopy (SEM), or X-ray computed tomography (XCT) [22]. In this work, the interpretation of the test results is restricted to a phenomenological approach.

The degree to which late hydration proves to be beneficial for the shear load bearing capacity of TRM/concrete joints undergoing repetitive freezing–thawing depends upon a multitude of parameters; the most important one is the matrix microstructure. The matrices employed in this work—with the exception of the sand used—share the same constituent materials but different mix proportions. In order to compensate for strength loss due to the use of pumice sand, a stronger (denser) paste (with a lower water-to-cementitious material ratio than mortar N) was designed for the lightweight mortar. It must be remembered that the basis of the comparison of the mortars was their nominal compressive strength. Dense pastes tend to inhibit water ingress and slow down the late hydration rates.

As previously described, the frost damage of the pumice-based mortars was heavier than the respective damage to limestone ones. This observation might seem contradictory to the common perception that lightweight matrices comprising porous aggregates (with an open porous system) outperform their normal weight counterparts under freeze–thaw conditions. Nevertheless, this perception is true when the basis of comparison is the mix designs of the mortars. Benefits are indeed recorded when fractions of the total of normal weight aggregates are replaced with lightweight ones (accompanied by an unavoidable penalty in strength). However, this is not without a limit. Ref. [23] reported that the freeze–thaw durability of a lightweight carbon fiber reinforced cement composite increases with increasing lightweight aggregate content up to a certain limit. Higher aggregate contents infer negative effects to the freeze–thaw durability of the composite.

The difference in the *σ_max_* values between the TRNM and TRLM specimens subjected to 70 freeze–thaw cycles shows the difference in the shear bond capacity of freeze–thaw-stricken mortar/concrete interfaces when the mortar changes from normal-weight to lightweight. This means that the lightweight mortar/concrete interface exhibits superior freeze–thaw resistance to the respective normal-weight mortar/concrete one without excluding the possibility that the former is also intrinsically better than the latter, even in the absence of detrimental exposure environments, such as freeze–thaw environments. Indeed, the internal curing provided by the water absorbed by the lightweight aggregates is believed to have led to a better chemical bond between the lightweight cementitious matrix and the concrete substrate. The late hydration of the lightweight mortar seemed to compensate for matrix frost damage from up to 60 cycles of freeze–thaw exposure. The outlying behavior of the FT70MLi specimens (in terms of the shape of the stress–strain curve and *σ_max_*) is due to the enhancement of the textile-to-matrix bond conditions, which, in turn, can be attributed to the slow rate of late hydration. Therefore, the benefits of the latter become more prominent at a longer exposure duration compared to the TRNM specimens.

## 5. Conclusions

The current study was dedicated to the experimental investigation of the effect of freeze–thaw cycling on the TRM-to-concrete bond, given the fact that previous related studies on the topic are extremely limited. The applied composite material consisted of a carbon dry fiber textile between two matrix layers. Two mortar types were used as matrix, a normal-weight and a lightweight one, both containing the same hydraulic powders but different aggregates, i.e., limestone and pumice sand, respectively. The single-lap/single-prism set up was applied after the hydro-thermal treatment of the specimens for various numbers of freeze–thaw cycles (according to ASTM C 666-Procedure B [7]).

- All of the TRNM specimens (except for the ones subjected to 70 freeze–thaw cycles), control TRLM specimens, and TRLM specimens subjected to 10 freeze–thaw cycles failed due fiber rupture. The rest of the TRLM specimens (except for the ones subjected to 70 freeze–thaw cycles) failed due to the slippage of the textile from the mortar. This failure mode was also accompanied by the rupture of (a small number of) individual sleeve fibers. All of the specimens subjected to 70 freeze–thaw cycles failed due to the TRM debonding from the substrate.
- Two damage paths were identified: one through the textile protruding from the mortar both at the loaded and the free end and another through the top mortar layer.
- The damage processes did not have an adverse effect on the *σ_max_* values for all of the specimens subjected to up to 60 freeze–thaw cycles. The proposed explanation for this phenomenon can be the late hydration of the mortars, which was in dynamic antagonism with those of frost heaving.
- The frost damage of pumice-based mortars was heavier than the respective damage of limestone ones that had undergone up to 60 cycles. However, the benefits of late hydration became more prominent at longer exposure durations for the TRLM specimens in comparison with the TRNM ones (70 cycles), which was probably due to the slower late hydration evolution rate in the lightweight matrix.

The current topic is offered for future work since the existing experimental results are extremely limited. In order for this composite material to be developed as an externally applied protective overlay of concrete elements exposed to freeze–thaw conditions, more parameters have to be investigated, such as the number of the TRM overlays as well as the constituent material type and mechanical characteristics.

## Figures and Tables

**Table 1:** Composition and physical properties of matrices.

Matrix	Normal-Weight	Lightweight
Composition		
Portland cement (CEM II 42.5N)	586 kg/m^3^	610 kg/m^3^
Sand (*d_max_* = 2 mm) *	1024 (limestone) kg/m^3^	550 (pumice) kg/m^3^
Silica fume (*d_max_* = 1 μm)	47 kg/m^3^	49 kg/m^3^
Limestone filler (*d_max_* = 120 μm)	146 kg/m^3^	152 kg/m^3^
Effective water **	344 kg/m^3^	316 kg/m^3^
Water to cementitious materials	0.54	0.48
Air content	1.9% (by vol.)	1.2% (by vol.)
Fresh mortar flow value [16]	14.5 mm	13.7 mm

* Water absorption: 3% and 22.28% by dry sand mass for the limestone and the pumice sand, respectively, ** The water quantity was adjusted during mixing beyond the effective one based on the water absorption and the moisture content of each type of sand.

**Table 2:** Experimental results.

Specimen	Number of Freeze–Thaw Cycles	*σ_max_* (MPa)	*σ_max,average_* (MPa) {CoV}	*d_r,max_* (mm)	*d_r,max,average_* (mm) {CoV}	*s_max_* (mm)	Failure Mode *
FT0MN01	0	457.95	486.71	0.45	-	0.00	FR
FT0MN02	0	515.47	{8%}	- **	-		FR
FT10MN01	10	459.29	521.14	0.37	0.49	0.01	FR-PD
FT10MN02	10	582.99	{17%}	0.61	{35%}		FR-PD
FT30MN01	30	489.20	494.43	0.49	0.62	0.10	FR-PD
FT30MN02	30	499.66	{1%}	0.75	{30%}		FR-PD
FT50MN01	50	554.96	595.70	- **	-	0.08	FR-PD
FT50MN02	50	636.44	{10%}	0.85	-		FR-PD
FT60MN01	60	641.73	627.76	1.43	1.20	0.08	FR-PD_PIC_
FT60MN02	60	613.79	{3%}	0.98	{27%}		FR-PD_PIC_
FT70MN01	70	176.18	157.23	0.07	0.08	0.01	D_PIC_
FT70MN02	70	138.28	{17%}	0.09	{19%}		D_PIC_
FT0ML01	0	524.72	533.31	- **	-	0.02	FR
FT0ML02	0	541.91	{2%}	0.46	-		FR
FT10ML01	10	539.18	549.25	0.37	0.45	0.01	FR-PD
FT10ML02	10	559.32	{3%}	0.53	{25%}		FR-PD
FT30ML01	30	511.29	479.72	- **	-	0.21	TS-PD
FT30ML02	30	448.15	{9%}	0.72	-		TS-PD
FT50ML01	50	490.57	504.24	0.92	0.84	0.36	TS-PD_PIC_
FT50ML02	50	517.90	{4%}	0.77	{13%}		TS-PD_PIC_
FT60ML01	60	576.17	567.03	1.44	1.70	0.64	TS-PD_PIC_
FT60ML02	60	557.89	{2%}	1.95	{21%}		TS-PD_PIC_
FT70ML01	70	820.12	778.08	2.13	2.00	0.12	D_PIC_
FT70ML02	70	736.05	{8%}	1.87	{9%}		D_PIC_

* FR = fiber rupture, TS = textile slippage, PD = partial detachment, PIC = pre-existent interlaminar crack, D = TRM debonding, ** Non-reliable records due to out-of-plane rotation of the aluminum plate during testing.

## Data Availability

Data sharing not applicable.

## References

[1] Raoof S.M., Koutas L.N., Bournas D.A. (2016). Bond between textile-reinforced mortar (TRM) and concrete substrates: Experimental investigation. Compos. Part B Eng..

[2] D’Ambrisi A., Feo L., Focacci F. (2013). Experimental analysis on bond between PBO-FRCM strengthening materials and concrete. Compos. Part B Eng..

[3] Al-Lami K., D’Antino T., Colombi P. (2020). Durability of Fabric-Reinforced Cementitious Matrix (FRCM) Composites: A Review. Appl. Sci..

[4] Yin S.-P., Li Y., Jin Z.-Y., Li P.-H. (2018). Interfacial Properties of Textile-Reinforced Concrete and Concrete in Chloride Freezing-and-Thawing Cycle. ACI Mater. J..

[5] Al-Jaberi Z., Myers J.J., Chandrashekhara K. (2018). Effect of direct service temperature exposure on the bond behavior between advanced composites and CMU using NSM and EB techniques. Compos. Struct..

[6] Colombo I.G., Colombo M., di Prisco M. (2015). Tensile behavior of textile reinforced concrete subjected to freezing–thawing cycles in un-cracked and cracked regimes. Cem. Concr. Res..

[7] ASTM International (2015). ASTM C666: Standard Test Method for Resistance of Concrete to Rapid Freezing and Thawing.

[8] Machovec J., Reiterman P. (2018). Influence of aggressive environment on the tensile properties of textile reinforced concrete. Acta Polytech..

[9] Nobili A., Signorini C. (2017). On the effect of curing time and the environmental exposure on impregnated Carbon Fabric Rein-forced Cementitious Matrix (CFRCM) composite with design considerations. Compos. Part B Eng..

[10] de Munk M., el Kadi M., Tsangouri E., Vervloet J., Verbruggen S., Wasties J., Tysmans T., Remy O. (2018). Influence of environmental loading on the tensile and cracking behavior of textile reinforced cementitious composites. Constr. Build. Mater..

[11] Belgisch instituut voor normalisatie (BIN) (2007). NBN EN 12467 Fibre Cement Flat Sheets—Product Specification and Test Methods, Toelating.

[12] Yin S., Jing L., Yin M., Wang B. (2018). Mechanical properties of textile reinforced concrete under chloride wet-dry and freeze-thaw cycle environments. Cem. Concr. Compos..

[13] Yin S.-P., Na M.-W., Yu Y.-L., Wu J. (2017). Research on the flexural performance of RC beams strengthened with TRC under the coupling action of load and marine environment. Constr. Build. Mater..

[14] CEN (1999). EN ISO 13934-1: Textiles-Tensile Properties of Fabrics–Part 1: Determination of Maximum Force and Elongation at Maximum Force Using the Strip Method.

[15] CEN (1993). EN 1015-11: Methods of Test for Mortar for Masonry–Part 11: Determination of Flexural and Compressive Strength of Hardened Mortar.

[16] CEN (1999). EN 1015-3 + A1: Methods of Test for Mortar for Masonry–Part 3: Determination of Consistence of Fresh Mortar (by Flow Table) (Includes Amendment A1:2004).

[17] International Code Council Evaluation Services (2013). AC434 ICC-ES: Masonry and Concrete Strengthening Using Fiber-Reinforced Cementitious Matrix (FRCM) Composite Systems.

[18] D’Antino T., Gonzalez J., Pellegrino C., Carloni C., Sneed L.H. (2016). Experimental Investigation of Glass and Carbon FRCM Composite Materials Applied onto Concrete Supports. Appl. Mech. Mater..

[19] Askouni P.D., Papanicolaou C.G. (2017). Experimental investigation of bond between glass textile reinforced mortar overlays and masonry: The effect of bond length. Mater. Struct..

[20] Sabau C., Gonzalez-Libreros J.H., Sneed L.H., Sas G., Pellegrino C., Täljsten B. (2017). Use of image correlation system to study the bond behavior of FRCM-concrete joints. Mater. Struct..

[21] De Felice G., Aiello M.A., Caggegi C., Ceroni F., De Santis S., Garbin E., Gattesco N., Hojdys Ł., Krajewski P., Kwiecień A. (2018). Recommendation of RILEM Technical Committee 250-CSM: Test method for Textile Reinforced Mortar to substrate bond characterization. Mater. Struct..

[22] Sokhansefat G., Moradian M., Finnell M., Behravan A., Ley M.T., Lucero C., Weiss J. (2020). Using X-ray computed tomography to investigate mortar subjected to freeze-thaw cycles. Cem. Concr. Compos..

[23] Soroushian P., Nagi M., Okwuegbu A. (1992). Freeze−Thaw durability of lightweight carbon fiber reinforced cement composites. ACI Mater. J..

